# Sustainable Involvement of Family Physicians for Improving Help-Seeking Behaviors in Rural Communities: A Thematic Analysis

**DOI:** 10.7759/cureus.50740

**Published:** 2023-12-18

**Authors:** Ryuichi Ohta, Toshihiro Yakabe, Chiaki Sano

**Affiliations:** 1 Community Care, Unnan City Hospital, Unnan, JPN; 2 Family Medicine, Unnan City Hospital, Unnan, JPN; 3 Community Medicine Management, Shimane University Faculty of Medicine, Izumo, JPN

**Keywords:** help seeking behaviors, cultural competency, community participation, health behavior, physician-patient relations, rural health services

## Abstract

Introduction

Medicine, particularly family medicine, is crucial to community health and well-being. Its impact on sustainable community care is significant, especially in rural settings with unique dynamics. Recent trends highlight the need for collaboration between family physicians and community members to foster effective help-seeking behaviors (HSBs) linked to improved quality of life and self-efficacy in self-management. This study explores rural communities' perceptions of community care and the integration of family physicians into rural healthcare, enhancing its sustainability.

Method

A thematic analysis based on relativist ontology and constructivist epistemology was employed. The study was conducted in Unnan City, Japan, involving 81 rural community members. Focus group interviews were the primary data collection method. The research team, comprising a family physician, a non-profit organization director, and a medical educator, analyzed the data, ensuring a balanced and unbiased approach.

Results

Four key themes emerged, such as understanding the burden felt by existing organizations, continuously engaging in meaningful community activities, steady activities matched to the community’s pace, and viewing all places as opportunities for exchange and learning. These themes reflect the need for better information sharing, respecting community dynamics, and incorporating family physicians into various community interactions for effective healthcare delivery.

Conclusion

This study emphasizes the critical role of family physicians in rural healthcare. It identifies the need for meaningful engagement with local communities, adapting healthcare to the rural context, and using various community spaces for health education. The findings advocate for a community-centric healthcare approach, which respects the unique dynamics of rural areas, fostering a sustainable and responsive healthcare system. Future research should include diverse rural settings and quantitative methods for broader applicability and deeper insights.

## Introduction

Medicine is a cornerstone of community health and well-being, playing a crucial role in shaping the health status of communities. This is particularly true in family medicine, where its impact on sustainable community care is paramount [1,2]. Recent trends indicate that a simplistic, top-down medical approach is insufficient. Instead, there is a growing need for robust collaboration between family physicians and community members to foster effective help-seeking behaviors (HSBs) [3,4]. HSB is defined as actions of acting by themselves or seeking help from various services or people in their communities. Previous studies have linked effective HSBs with improved quality of life, particularly in terms of self-management self-efficacy [5]. Enhancing HSBs is particularly relevant in rural settings, which have unique dynamics.

Rural communities, with their distinct cultures, traditions, and beliefs, have specific perceptions of community care sustainability [6]. Effective healthcare in these areas involves not only the provision of medical services but also a deep understanding and respect for these unique cultural nuances [7]. Previous research in rural settings indicates that ongoing dialogue between family physicians and community members can strengthen relationships, enhancing HSBs [8,9]. An integrated approach, where family physicians actively engage in rural healthcare, understand local culture, and utilize this knowledge, can significantly improve HSBs.

However, there still needs to be a knowledge gap regarding how rural communities perceive community care, their expectations, fears, and hopes. Furthermore, the practical and effective integration of family physicians into rural healthcare, ensuring sustainability, remains unclear. This research aims to explore these aspects, shedding light on rural communities' perceptions of community care. It seeks to propose practical strategies for integrating family physicians into the rural healthcare framework, thereby enhancing its long-term sustainability.

## Materials and methods

This study adopted a thematic analysis rooted in relativist ontology and constructivist epistemology to understand the complex social and cultural backgrounds influencing rural healthcare perceptions. We aimed to explore how these perceptions, varying across different contexts and backgrounds, affect the engagement of family physicians in enhancing HSBs in rural areas. The analysis was anchored in a qualitative framework, focusing on the nuances of rural community care and physician involvement [10].

Setting

The research was conducted in Unnan city, located in Shimane Prefecture, Japan. This city, encompassing 553.1 km², is characterized by its forest-covered landscape and rural setting. A 2020 survey highlighted its aging population, with 40.01% over 65 years, within a total population of 36,007. The city's social structure is organized into 30 multifunctional autonomous communities, each addressing specific social needs and issues [11].

Participants

From April to September 2023, 81 rural community members from Unnan City participated in discussions about family physicians' role in rural healthcare. Participants included local stakeholders like non-profit organization directors, local lawmakers, healthcare workers, and residents. We employed purposive sampling for focus group interviews, targeting individuals over 18 years capable of providing informed consent. Exclusion criteria included non-residents and those unable or unwilling to participate.

Data collection

Focus Group Interviews

Focus group interviews were the primary data collection method. Held weekly in Unnan City Hospital's conference room, these groups ranged from three to six participants. Discussions, facilitated by the second author (TY) and noted by the first author (RO), focused on potential collaborations between family physicians and citizens. These sessions, lasting 50-70 minutes, were recorded and transcribed for analysis.

Reflexivity

The research process was profoundly shaped by the diverse backgrounds and expertise of the research team, emphasizing a collaborative interaction between the researchers and participants.

The team comprised a family physician and public health professional (RO), a director of a non-profit organization experienced in rural community support (TY), and a medical educator specializing in community healthcare management (CS). Each member brought a unique perspective: RO, with a master's degree in public health and family medicine and hands-on experience in rural community health; TY's two decades of work in supporting isolated individuals in communities offered grassroots understanding of rural dynamics; CS, as a medical educator and professor, contributed an academic and systematic approach to community health care management and education.

To ensure a balanced and unbiased approach, the team engaged in rigorous discussions, continually challenging and refining each other's ideas and interpretations. This included exploring alternative viewpoints and considering the influence of their professional and personal experiences on the data analysis. This reflective practice was crucial in mitigating potential biases and assumptions, ensuring a more objective and comprehensive understanding of the study findings.

The researchers maintained a respectful and empathetic engagement with the participants, acknowledging the value of their insights and experiences. This participatory approach facilitated a deeper understanding of the nuances in rural healthcare perceptions and practices.

Analysis

The analysis employed an in-depth inductive thematic approach, meticulously examining the data to uncover underlying themes related to social isolation, health problems, and solutions within rural contexts [10].

Interviews were recorded, transcribed verbatim, and then subjected to a thorough analysis. Following each set of three interviews, RO and TY independently read the transcripts, coding them to identify emerging patterns and themes. This process involved iterative readings, with each reading providing a deeper understanding and refinement of the data.

Initially, RO developed a preliminary codebook, which was continually revised based on the transcripts. TY, in parallel, independently reviewed the transcripts, contributing to the coding process. The two researchers then conducted comprehensive discussions, comparing and contrasting their findings, merging codes, and refining themes. This collaborative coding process ensured a multiperspective approach to data interpretation.

The iterative process of coding and discussion continued until no new themes emerged and a mutual consensus was reached on the final set of themes. In this final stage, the involvement of CS, the community care specialist, added an additional layer of expertise, validating the themes and ensuring their relevance to community care practices.

Once the themes were finalized, the results were translated from Japanese into English, ensuring the nuances and context of the original data were preserved. This translation process was crucial for accurately conveying the findings to a broader, international audience.

Ethical consideration

The Unnan City Hospital Clinical Ethics Committee approved the study protocol (no.: 20230024).

## Results

Results of the thematic analysis

The thematic analysis identified four key themes concerning the perceptions of rural populations about community care. It proposed practical methods for integrating family physicians into the rural community care framework to enhance its long-term sustainability (Table 1).

**Table 1:** The result of the thematic analysis

Theme	Explanation
Understanding the burden felt by existing organizations	There was a strong sense of resistance among community organizations toward the increasing workload imposed by local governments, which was perceived as burdensome and not necessarily beneficial to the community. With growing responsibilities, especially around personal information management, there was a decline in community engagement. A drop in local interactions and weakening relationships was noted. The organizations expressed a desire to focus on activities unique to them, rather than substituting for public services. Strategies for better information sharing within the community, especially for the elderly, were considered necessary.
Continuously engaging in meaningful community activities	The analysis found that the absence of personal relationships impeded immediate consultation. Building and maintaining relationships between organizations was essential. Acknowledging the diversity of individuals involved in community activities and respecting different generations were highlighted as important. Regular dialogue and planning with all stakeholders were necessary to address the generational gap and improve community health outcomes.
Steady activities matched to the community’s pace	Resistance to rapid external changes was common. Steady relationship building, tailored to the unique pace of each community and gradual advancement of activities, was emphasized. A methodical approach to health-related community activities and patience in engagement was seen as vital. The need to respect each community's cultural, historical, and relational specifics when sharing health-related information was stressed.
Viewing all places as opportunities for exchange and learning	Family physicians should venture into various locations, not limited to specific venues, to increase the affinity with local hospitals and understand community needs better. It was suggested that information on healthcare behaviors could be disseminated through personal gatherings and that family physicians should be accessible for questions in designated facilities. The importance of dialogue with community health workers was underscored, suggesting that they could gather information on local issues and share it with family physicians via social media, which might lead to smoother medical responses in the community.

**Figure 1:**
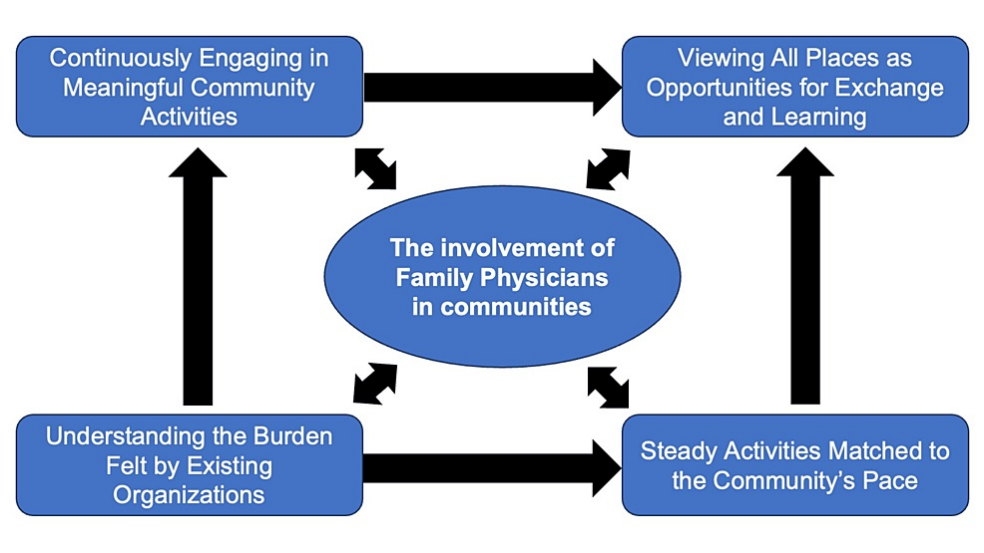
The conceptual figure for the perceptions of rural populations regarding community care proposing tangible methods for integrating family physicians in rural community care

The conceptual framework is illustrated in Figure 1.

Understanding the burden felt by existing organizations

The research identified a significant resistance among rural stakeholders to an increasing number of tasks imposed by local governments, which were perceived as more burdensome than beneficial. This resistance was articulated by Participant 3 (a 67-year-old man) who stated, “I felt a burden from the local government. The government asks for various activities in communities. However, most suggestions may not be meaningful for us, just their duty of their jobs.” Similarly, Participant 2 (a 75-year-old woman) expressed a reluctance to engage in government-enforced activities due to time constraints, noting, “I don’t do enforced activities from the local government because of limited time. The lack of time may make the distance for new activities for communities.” This sentiment reveals a fundamental disconnect between the local government’s expectations and the community’s capacity and willingness to comply.

Furthermore, the study highlighted a general consensus among participants that tasks involving personal information management, typically a governmental responsibility, are seen as highly burdensome. This has led to a notable decline in community engagement and interaction. Participant 14 (an 81-year-old man) underscored the challenges related to privacy and personal information issues in healthcare: “In present societies, privacy and personal information issues are critical. I am also a layperson regarding healthcare, so I cannot approach isolated people without knowing their conditions.” Participant 11 (a 71-year-old man) expressed a desire for community support while avoiding entanglement in complex issues, emphasizing a preference for a simpler, more sustainable community life: “I want to support and be supported by others for the sustainability of rural communities. However, I prefer not to become entangled in various issues...”

Continuously engaging in meaningful community activities

The study found that community organizations focused on unique tasks specific to their capabilities rather than assuming responsibilities typically managed by public organizations. Participant 11 (a 70-year-old woman) encapsulated this sentiment: “Community organizations should not be substitutions for governments. There are various things specific for community organizations.” This preference stems from a desire to effectively address community-specific needs, particularly in the face of increasing isolation and challenges in information dissemination. Participant 4 (a 75-year-old man) emphasized the importance of supporting isolated individuals through community relationships: “We have to consider privacy and personal information issues. However, various isolated people can be supported by each community’s relationship...”

The research also underscored the importance of engaging family physicians in healthcare initiatives and respecting and leveraging existing relationships within the rural community. Participant 7 (a 56-year-old woman) highlighted the positive reception of family physicians in community gatherings: “Present gatherings should be respected and used for healthcare improvement by family physicians. Rural people welcome them at various private houses gathering indigenous older people.” Additionally, Participant 23 (a 78-year-old man) noted the need for continuous relationship building between organizations and healthcare providers to facilitate effective interventions and respect diverse opinions within the community: “Building the relationship is essential for effective interventions. Family physicians should continuously participate in community activities to establish the relationship with indigenous people, respecting different opinions in communities.”

Steady activities matched to the community’s pace

Participants emphasized the need for a steady and gradual approach to community activities, aligned with each rural community's unique pace and cultural context. Participant 1 (an 84-year-old man) highlighted the disparity in change pace: “The difference of changing speed should be respected. Rural communities cannot change themselves smoothly.” This observation suggests a need for healthcare initiatives to be more attuned to the gradual pace of change inherent in rural settings. Participant 7 (a 60-year-old woman) stressed the importance of mutual understanding and dialogue between healthcare professionals and community members for effective health promotion: “Through continual dialogue among them, effective health promotion can be possible.”

The study highlighted the necessity of a systematic, culturally sensitive approach to health promotion, built through dialogue and careful assessment. Participant 20 (a 76-year-old man) emphasized the importance of family physicians’ involvement in rural community health promotion, advocating for a continuous and steady approach: “The involvement of family physicians in rural community health promotion is vital for the sustainability of communities. I hope that their approaches to communities should be steady and continuous, not transient.” Participant 31 (a 70-year-old woman) further mentioned the importance of respecting existing relationships and histories within rural communities: “The respect for previous relationships should also be respected. There are various relationships among rural citizens affected by their histories.”

Viewing all places as opportunities for exchange and learning

The research highlighted the potential of viewing all community spaces as platforms for exchange and learning. Participant 9 (a 50-year-old man) observed the natural congregation of rural people in informal settings and the impact of such gatherings on health behaviors: “Rural people usually meet each other by gathering at someone’s house and have dialogues about their usual issues including health conditions. In the dialogues, they may change their health behaviors.” This insight underscores the opportunity for family physicians to participate in such gatherings, fostering a deeper understanding of community health needs.

The role of community health workers in facilitating information exchange was also emphasized. Participant 11 (a 60-year-old man) suggested using social media to communicate effectively between rural citizens and physicians: “Social media can be beneficial for information sharing among citizens and physicians...” This approach could lead to more efficient and responsive rural healthcare. Participant 13 (a 67-year-old woman), a community center worker, noted the potential for direct communication with family physicians to address health issues effectively: “I work at a community center and know various difficulties in health among citizens. I hope to communicate with family physicians regarding such issues and get suggestions for mitigating the difficulties.”

## Discussion

This study explored the perceptions of rural populations regarding community care and highlighted potential strategies for integrating family physicians into the rural community care framework. The findings revealed four major themes: (1) understanding the burden felt by existing organizations, (2) continuously engaging in meaningful community activities, (3) steady activities matched to the community’s pace, and (4) viewing all places as opportunities for exchange and learning.

The study highlighted the resistance among community organizations toward the increasing workload imposed by local governments. This resistance suggests a need to reevaluate the roles and responsibilities designated to these organizations [12,13]. Our findings echo the sentiments of the stakeholders, who articulated the burden of governmental demands [14]. As the stakeholders indicated, this burden leads to a decline in community engagement, emphasizing the importance of roles that respect the capabilities and limitations of these organizations. The previous articles in rural contexts clarified that rural citizens needed help establishing effective relationships with local governments because of the need for mutual understanding of standpoints [15,16]. For effective community organizing, rural communities and local governments should have dialogues regarding their roles in community sustainability, including family physicians [17,18].

The findings of the desire to continuously engage in meaningful community activities underline the significance of maintaining and nurturing personal relationships within the community as stated by the rural stakeholders in this study. As previous research shows, community organizing and empowered activities in rural communities can be possible when rural citizens and stakeholders realize their activities are meaningful for their communities [19]. For the continuity of effective community organizing, dialogue and regular planning, especially with older community members, become crucial for health information dissemination and community engagement as highlighted by the rural stakeholders. As previous research shows, the continuity of family physicians’ involvement in community activities is essential for citizens’ motivations [20]. The relationship among them can be established through continuity, which can drive their activities effectively [21]. These findings suggest that rural community organizing and family physician involvement should be continual and constantly progressive through the dialogue among them.

Steady activities matched to the community’s pace can be one of the tips for continual and progressive community approaches to family medicine. This study's key findings were the resistance to rapid external changes and the need for a gradual, tailored approach to community activities. The rural stakeholders emphasize the importance of respecting the unique pace and cultural specifics of each community. In the management of organizations, the change in organizations demands a long duration for the acceptance of members in communities and moderation of resistance to change, which can be accented by sociocultural contexts [22]. Especially, rural contexts have a strongly established culture by local people continuously [23]. This study suggests a methodical approach to health-related activities, underlining the need for patience and understanding in community engagement. For family medicine, effective community approaches should be based on this theme and pace the involvement in their communities through dialogues with rural citizens and stakeholders.

Viewing all places as opportunities for exchange and learning health information can be beneficial in rural communities. In this study, the participants’ insights suggest that family physicians should be more proactive in community involvement. The outreach of family physicians can vary from local community centers to citizens’ homes [20]. One of the previous studies in rural contexts shows that family physicians’ involvement in rural community’s health promotion can motivate citizens to revise and improve their help-seeking behaviors [8]. In addition, some stakeholders’ observation about the role of personal gatherings in influencing health behaviors highlights the potential of these interactions in promoting health education and awareness driven by family physicians [24]. This finding shows the importance of any gathering involving family physicians in improving rural citizens’ healthcare. Further, the use of social media, as suggested by rural stakeholders, could enhance the communication between community health workers and family physicians [25,26]. This finding can be supported by previous social medical articles, so even in rural areas, social media should be used for older people’s health issues supported by their families and community workers [27,28].

The study’s findings advocate for a community care framework that is more aligned with the needs, pace, and cultural contexts of rural populations. It underscores the importance of building and sustaining relationships, respecting the unique dynamics of rural communities, and utilizing a variety of platforms for health communication and education [29]. The integration of family physicians into this framework should be thoughtful and patient, reflecting the community's pace and preferences [30].

This study has limitations. Its focus on specific rural communities may limit the generalizability of the findings to other rural or urban settings. The reliance on self-reported data from participants' and interviewers' characters and backgrounds could introduce biases and may not fully capture the complexity of community care dynamics. Additionally, the study's qualitative nature restricts quantitative analysis of the impact of proposed interventions. Future research should include a more diverse range of rural settings and incorporate quantitative methods to validate and expand upon these findings such as questionnaires measuring their perceptions of community involvement.

## Conclusions

This study underscores the importance of family physicians in rural healthcare, focusing on four main areas: the increasing burdens on community organizations, the need for meaningful engagement with local communities, adapting healthcare to the rural context, and the use of community spaces for health education. It highlights the importance of balanced task distribution and cultivating relationships, particularly with the elderly. Tailored, culturally sensitive healthcare interventions are emphasized. The study advocates for family physicians' active involvement in community activities with continuous dialogues for better HSBs and effective health communication and provision of various health promotion information through digital platforms. Overall, it calls for a community-centric healthcare approach that understands and responds to the unique dynamics of rural areas, fostering a more sustainable and responsive healthcare system.
